# High deoxynivalenol and ergot alkaloid levels in wheat grain: effects on growth performance, carcass traits, rumen fermentation, and blood parameters of feedlot cattle

**DOI:** 10.1007/s12550-024-00534-5

**Published:** 2024-05-03

**Authors:** R. M. Bierworth, G. O. Ribeiro, S. A. Terry, N Malmuthuge, G. B. Penner, J. J. McKinnon, P. Hucl, H. Randhawa, K. A. Beauchemin, K. Stanford, K. Schwartzkopf-Genswein, W. Z. Yang, R. Gruninger, L. L. Guan, D. Gibb, T. A. McAllister

**Affiliations:** 1 Department of Animal and Poultry Science, College of Agriculture and Bioresources, University of Saskatchewan, Saskatoon, SK S7N 5A8 Canada; 2 Agriculture and Agri-Food Canada, Lethbridge Research and Development Centre, Lethbridge Alberta, T1K 4B1 Canada; 3 Department of Agricultural Food and Nutritional Science, Faculty of Agricultural, Life, and Environmental Sciences, University of Alberta, Edmonton, AB T6G 2R3 Canada; 4 Department of Biological Sciences, University of Lethbridge, Alberta, T1K 3M4 Canada; 5 Gowan’s Feed Consulting, Raymond, AB T0K 2S0 Canada

**Keywords:** Deoxynivalenol, Ergot alkaloids, Beef cattle, Wheat grain

## Abstract

This study was designed to assess the impacts of a mixture of deoxynivalenol (DON) and ergot alkaloids (EAs) on growth performance, rumen function, blood parameters, and carcass traits of feedlot cattle. Forty steers (450 ± 6.0 kg) were stratified by weight and randomly allocated to 1 of 4 treatments; control-low (CON-L), control-high (CON-H) which contained low or high wheat screenings that lacked mycotoxins at the same level as the mycotoxin-low (MYC-L; 5.0 mg/kg DON, 2.1 mg/kg EA), and mycotoxin-high (MYC-H: 10 mg/kg DON, 4.2 mg/kg EA) diets that included wheat screening with mycotoxins. Steers were housed in individual pens for a 112-day finishing trial. Intake was 24.8% lower (*P* < 0.001) for MYC steers compared to CON steers. As a result, average daily gains of MYC steers were 42.1% lower (*P* < 0.001) than CON steers. Gain to feed ratio was also lower (*P* < 0.001) for MYC steers compared to CON steers. Platelets, alanine aminotransferase, globulins, and blood urea nitrogen were lower (*P* ≤ 0.008), and lymphocytes, glutathione peroxidase activity (GPx), and interleukin-10 (IL-10) were elevated (*P* ≤ 0.002) in MYC steers compared to CON steers. Hot carcass weights and backfat thickness were reduced (*P* < 0.001) in MYC steers, resulting in leaner (*P* < 0.001) carcasses and higher (*P* < 0.007) meat yield compared to CON steers. Results suggest that a mixture of DON and EAs negatively impacted health, performance, and carcass traits of feedlot steers, with the majority of this response likely attributable to EAs. However, more research is needed to distinguish the relative contribution of each mycotoxin to the specific responses observed.

## Introduction

Understanding the implications of environmental shifts due to climate change on agriculture has become a topic of concern not only in Canada, but globally. The warmer seasonal temperatures and increased precipitation during spring in western Canada (Erler and Peltier 2016) has increased the incidence of *Fusarium graminearum* head blight (FHB) and *Claviceps purpurea* (ergot). These fungi produce several mycotoxins that can have deleterious effects not only on the crop, but also on livestock and humans (Döll and Dänicke 2011; Coufal-Majewski et al. 2016). The most predominant mycotoxins produced by these species include deoxynivalenol (DON) and ergot alkaloids (EAs), respectively. Contamination of cereal crops with these pathogens has been the leading cause of cereal grain downgrading (He et al. 2015), with wheat being only second to rye in the level and frequency of FHB and ergot contamination (Walkowiak et al. 2022; Bamforth et al. 2022). Increasingly, wheat grain that fails to meet milling standards is utilized as feed for feedlot cattle (Saleem et al. 2022). Although caution must be taken to mitigate the occurrence of digestive upset like acidosis and bloat in cattle fed wheat, proper adaptation can enable it to be fed up to 90% of total diet dry matter (DM) without complications (He et al. 2015).

If the cost of contaminated wheat is lower than other grains, its use can also increase profitability, provided that it does not have a detrimental impact on growth performance or health. Ruminants can tolerate higher dietary levels of DON than most monogastrics, but are more sensitive to EAs than swine and poultry (Charmley et al. 1993; Coufal-Majewski et al. 2017). The ability of rumen microbiota to convert DON into its less toxic form, de-epoxy DON (DOM-1) is thought to be responsible for the lower sensitivity of ruminants to this mycotoxin (Seeling and Dänicke 2005; Döll and Dänicke 2011; Roberts et al. 2021). Research conducted by Charmley et al. (1993) showed that even at 12.1 mg DON/kg of DM, dairy cattle experienced no ill effects on milk production or feed intake. In contrast, Coufal-Majewski et al. (2017) found that compared to control lambs, lambs fed 2.45 mg/kg of EAs exhibited a linear decrease in ADG, gaining on average 256.9 g/day, while control lambs gained 353.6 g/day, with a 27% reduction in feed efficiency. A similar linear reduction in intake and ADG was observed more recently by Sarich et al. (2023) with beef cattle fed up to 3 mg/kg of EAs. Currently, Canadian regulatory limits are set at 5 mg/kg for DON and 2 to 3 mg/kg for EAs in beef cattle diets (DM basis, CFIA 2017a). With emerging research suggesting that DON regulations may be too stringent and EAs limits too lenient, both scenarios have the potential to effect animal health, welfare, and profitability. Consequently, the Canadian Food Inspection Agency (CFIA) is currently reviewing the mycotoxin tolerance levels for all livestock (CFIA 2017b).

While setting appropriate tolerance limits for individual mycotoxins in animal feed is important, these secondary metabolites rarely occur alone. Co-occurrence of mycotoxins in feed and their interactions are poorly defined, as well as their effects on humans and livestock. With little to no information available on mixtures of FHB and ergot, research is needed to further define their impact on the health and growth performance of feedlot cattle, even though the individual impact of each mycotoxin within a mixture on these parameters may not be ascertained. Therefore, the objective of this study was to evaluate the impact of two concentrations of DON and EAs in wheat-based diets on growth performance, rumen function, blood parameters, carcass quality, and the incidence of liver abscesses in feedlot steers.

## Methods and materials

This study was conducted at the Agriculture and Agri-Food Canada, Lethbridge Research and Development Centre (Lethbridge, AB, Canada). All procedures and protocols were reviewed and approved by the Lethbridge Research and Development Centre Animal Care Committee (Protocol #2015) with cattle cared for following guidelines of the Canadian Council on Animal Care (CCAC 2009).

### Wheat selection

Mycotoxin contaminated wheat screenings (MYC-wheat), and uncontaminated wheat screenings were procured through the industry from the University of Saskatchewan and sent to Prairie Diagnostic Services (Saskatoon, Saskatchewan, Canada) for mycotoxin testing using HPLC–MS/MS as per Biselli et al. (2005) and Monbaliu et al. (2009) for DON, and by Gruise et al. (2017) for EAs. Concentrations of DON and EAs in wheat grain and uncontaminated wheat screenings were below detectable levels. Sufficient wheat was purchased as a single lot so as to provide all of the grain needed for the entire experiment. Contaminated wheat screenings contained 16.2 mg/kg DON and 6.89 mg/kg EAs (DM basis) and were diluted with uncontaminated wheat screenings. Wheat grain was blended with MYC contaminated wheat screenings to generate a low mycotoxin diet (MYC-L, 5 mg/kg DON; 2.1 mg/kg EAs) that was within CFIA limits (CFIA 2017a), and a high mycotoxin diet (MYC-H, 10 mg/kg DON; 4.2 mg/kg EAs) that exceeded CFIA limits (5 mg/kg DON; 2–3 mg/kg EAs). Two control diets (CON-L, CON-H) were formulated with the same levels of uncontaminated wheat screenings relative to the MYC diets to ensure a similar nutritional profile between CON and MYC diets (Table 1).

**Table 1:** Chemical composition of finishing diets fed to beef steers (*n* = 4)

	Diet type			
Item	CON-L	CON-H	MYC-L	MYC-H
Diet ingredient (% of diet, DM^a^ basis)				
Wheat grain	52.00	20.00	52.00	20.00
Wheat screenings – MYC^b^	0.00	0.00	31.00	63.00
Wheat screenings	31.0	63.0	0.00	0.00
Barley silage	12.00	12.00	12.00	12.00
Supplement^c^	5.00	5.00	5.00	5.00
Chemical composition of TMR (% DM)				
Dry matter	81.8 ± 0.52	81.2 ± 0.73	80.3 ± 1.03	79.8 ± 1.43
Crude protein	17.3 ± 0.41	16.9 ± 0.57	16.9 ± 0.49	16.5 ± 0.71
Neutral detergent fiber	11.4 ± 0.90	13.5 ± 1.23	12.0 ± 0.97	13.9 ± 0.38
Acid detergent fiber	5.13 ± 0.49	6.40 ± 0.50	5.20 ± 0.80	6.73 ± 0.46
Starch	48.1 ± 4.22	43.6 ± 3.63	43.8 ± 3.58	42.0 ± 1.49
Deoxynivalenol, mg/kg	ND	ND	5.00	10.0
Ergot alkaloids, mg/kg	ND	ND	2.10	4.20

^a^DM: Dry matter

^b^Mycotoxin contaminated wheat

^c^Supplement contained (per kg) 572.02 g of ground barley, 250 g of calcium carbonate, 100 g of canola meal, 30 g of white salt, 25 g of molasses, 10 g of feedlot mineral, 10 g of canola oil, 2.32 g of Rumensin (Elanco Animal Health, Greenfield, IN; 25 mg of monensin/kg of DMI), and 0.66 g of vitamin E

### Animals, experimental design, and diets

Forty Angus crossbred steers (450 ± 37 kg), 8 ruminally cannulated and 32 intact were used in a 112-day finishing experiment. Steers were administered an implant containing 100 mg trenbolone acetate (TBA), 10 mg of estradiol, and 29 mg of tylosin tartrate (Component TE-100, Elanco Animal Health, Division Eli Lilly Canada Inc., Guelph, ON) upon arrival. They were also treated with Bovimectin Pour-on (5 mg of ivermectin/mL; 500 µg of ivermectin per kg of BW; Vetoquinol N.-A. Inc., Lavaltrie, QC) and vaccinated with Ultrabac 7/Somubac (Clostridium vaccine; Zoetis Canada Inc., Kirkland, QC), and Pyramid FP5 (infectious bovine rhinotracheitis and bovine viral diarrhea vaccine; Boehringer Ingelheim Ltd., Burlington, ON).

All steers were stratified by body weight and randomly assigned to one of the four treatments and housed in individual pens (5.2 × 1.8 m). This resulted in two ruminally cannulated steers and eight non-cannulated steers for each treatment. Steers fed MYC diets were stationed in separate but identical wings of the same barn as CON fed steers so as to prevent contamination across diets. All steers were provided with free access to fresh water via automatic waterers stationed at the front of each individual stall.

Steers were transitioned to finishing diets over a three-week period. Diets were prepared daily as a total mixed ration (TMR) and delivered to each stall using a Calan Super Data Ranger Mixer (American Calan Inc., Northwood, NH, USA). Control diets were fed first, followed by the diets with contaminated wheat screenings. The mixer was thoroughly cleaned before use on the following day. Steers were fed once daily at 0900 h to appetite using slick bunk feeding practices, targeting < 5% orts each day. Control diets were balanced to meet or exceed the nutrient requirements of finishing feedlot steers according to the National Academies of Sciences, Engineering, and Medicine (NASEM 2016) recommendations. Monensin (25 mg/kg of diet DM; Rumensin, Elanco Animal Health) was included in all diets.

### Feed and fecal analyses

Diets, silage, and orts were collected weekly, and individual feed ingredients (wheat grain, MYC-wheat grain, wheat screenings, and supplement) were collected monthly throughout the experiment. Each sample was analyzed for DM by drying at 55 °C for 72 h in a forced air oven. Weekly samples for diets and silage were composited for each 28-day period, while orts were not pooled and were stored as individual samples. Feed ingredient and TMR samples (*n* = 36) were ground through a 1-mm screen using a Wiley mill (Standard model 4 Wiley Mill; Arthur H. Thomas, Philadelphia, PA, USA) and sent to the Dairy One Forage Testing Laboratory (Dairy One Inc., Ithaca, NY) for analysis of DM, crude protein (CP), neutral detergent fibre (NDF), acid detergent fibre (ADF), and starch.

Analytical DM for feed samples was determined by drying samples at 135 °C for 2 h (AOAC 2005; method 930.15). Crude protein (CP = *N* × 6.25) was determined by combustion (method 990.03; AOAC 2005) using a CN628 or CN928 Carbon/Nitrogen Determinator (Leco Corporation, 300 Lakeview Avenue, St. Joseph, MI 49085). The NDF content was determined according to Van Soest et al. (1991) with heat-stable amylase and sodium sulfite used in the procedure (ANKOM Technology Method 6, Filter Bag Technique for A200). The ADF content was determined using an ANKOM DELTA digestion unit (ANKOM Technology, 2052 O’Neil Road, Macedon, NY 14502) (method 973.18; AOAC 2005). Starch content was determined by enzymatic hydrolysis (YSI 2700 select Biochemistry Analyzer, YSI, Yellow Springs, OH, USA; YSI Application Note 319) and consisted of pre-extraction in a 40 °C hot water bath followed by filtration, thermal solubilization via autoclaving and incubation with glucoamylase to produce dextrose (Herrera-Saldana et al. 1990).

Fecal grab samples were collected from all steers (*n* = 40) as they entered the chute during the monthly weighing. Samples were stored at − 20 °C and later composited by animal over the entire feeding trial. Fecal DM was determined by drying samples in a forced air oven at 55 °C for 72 h. Samples were then ground as described for feed and shipped to the department of Animal and Poultry Science laboratory (University of Saskatchewan, Saskatoon, SK, Canada) for analysis of starch, CP, NDF, ADF, lignin, and ash using near-infrared-spectroscopy (SpectraStar NearInfared analyzer 2400 RTW, Unity Scientific, Brookfeild, CT) as described by Jancewicz et al. (2016). By using the DM digestibility value and DMI from each pen, fecal output was then calculated. Digestibility of each nutrient was determined using fecal output, and the concentration of nutrients determined in diet and fecal samples.

### Ruminal pH and rumen contents

Ruminal contents were collected just prior to feeding (0700 to 0800 h) from the reticulum, ventral, caudal, and dorsal–ventral sac of the reticulorumen of each cannulated steer, prior to insertion of an indwelling pH meter (Dascor, Inc., Escondido, CA) into the ventral sac of the rumen of cannulated steers (*n* = 8). Prior to placement in the rumen, millivolt readings were used to calibrate loggers while the electrodes were submerged in pH 4 and pH 7 buffers. The pH loggers were placed in the rumen of steers just before feeding at 0800 h, one week prior to sample collection. Loggers measured pH every minute and were placed in the rumen at this time to enable their removal at the time of weighing. Ruminal pH data were summarized for daily average, minimum, maximum, and standard deviation (SD), as well as duration and area under the curve (AUC) at pH 6.0, 5.6, and 5.2 (Wierenga et al. 2010). The AUC was calculated as the sum of the absolute value of pH deviations below 6.0, 5.6, or 5.2, multiplied by the duration below pH 6.0, 5.6, or 5.2, respectively, and reported in minutes. Durations and AUC for pH 5.6 and 5.2 were used to describe the duration and severity of subacute ruminal acidosis (SARA) and acute ruminal acidosis (ARA), respectively. Intake-corrected measurements of AUC were calculated by dividing the AUC by DMI and reported as AUC per kg of DMI.

Ruminal contents from each region described above were combined, thoroughly mixed, and strained through 2 layers of Pecap nylon (pore size 355 µm; Sefar Canada Inc., Ville St. Laurent, QC, Canada). The pH of the collected rumen fluid was measured immediately using a portable pH meter. Two aliquots of rumen fluid (2.5 mL) were also collected and mixed with either 0.5 mL of 25% (wt/vol) metaphosphoric acid or 0.5 mL of 1% H_2_SO_4_ (vol/vol), for VFA and ammonia-N (NH_3_-N) analysis, respectively. For enumeration of protozoa, 2.5 mL of rumen fluid was mixed with 2.5 mL of a methyl green formalin-saline solution. Collected samples for VFA and NH_3_-N were stored at − 20 °C, whereas samples for protozoa counts were kept at room temperature in the dark prior to analysis. Volatile fatty acid concentrations were quantified using gas liquid chromatography (model 5890; Hewlett-Packard, Wilmington, DE). The chromatograph was equipped with a 30-mm Zebron free fatty acid phase fused silica capilliary, 0.32-mm i.d., and 1.0-µm film thickness (Phenomenex, Torrance, CA). Concentration of NH_3_-N in rumen samples was analyzed by the phenol-hypochlorite method as described by Broderick and Kang (1980). Protozoa were enumerated under a light microscope using a levy-Hausser counting chamber (Hausser Scientific, Horsham, PA, USA) with a 1-mm depth as described by Dehority (1993).

### Blood samples

Whole blood, serum, and plasma were collected from all steers via jugular venipuncture on each weigh day. Blood was collected into three separate evacuated tubes, one 10-mL tube containing sodium heparin (BD Vacutainer, Becton Dickinson Co., Franklin Lakes, NJ, USA) for plasma, one 10-mL tube without anti-coagulant for serum (BD Vacutainer, Becton Dickinson Co., Franklin Lakes, NJ, USA), and one 6-mL tube containing K_3_EDTA (Greiner Bio-One Vacuette, Greiner Bio-One Int., Monroe, NC, USA) for complete cell (CBC), red blood cell (RBC), white blood cell (WBC), platelet (PLT), and lymphocyte (LYM) counts. Both 10-mL tubes were centrifuged at 2000 × *g* for 20 min at 4 °C and the plasma was transferred to microtubes and stored at − 20 °C until analyzed. The samples collected in tubes containing K_3_EDTA were analyzed as whole blood samples to determine CBC values using a HemaTrue Hematology Analyzer (Heska, Loveland, CO, USA).

Harvested plasma was analyzed for aspartate aminotransferase (AST), alanine aminotransferase (ALT), gamma-glutamyl transpeptidase (GGT), albumin, globulins, and blood urea nitrogen (BUN) using a chemistry analyzer (VetTest analyzer, model 8008, IDEXX Lab, Westbrook, ME, USA). Harvested serum was used to estimate catalase and glutathione peroxidase (GPx) activities using colorimetric assays (Catalase—Assay kit ab83464, AbCam, Watham, MA; GPx—Assay kit ab102530, AbCam, Watham, MA, USA). Serum was also used to perform ELISAs for IL-6, IL-10, and TNF-α (IL-6 ELISA kit DIY0670B-003: IL-10 ELISA kit DIY2157B-003; TNF-α ELISA kit DIY0675B-003, Kingfisher Biotech, Inc., Saint Paul, MN, USA). All intra- and inter-assay CV values were ≤ 10% and ≤ 15% respectively.

### Hair samples

Hair samples were collected from the forehead of each steer during weighing by shaving as close to the skin as possible with clippers (Lister, Wahl Clippers Corp., Sterling, IL, USA). Hair samples were processed to determine cortisol concentrations as described by Moya et al. (2013). Briefly, hair was washed with 5 mL of 70% ethanol and dried at room temperature for 7 d prior to ball grinding (MM 400, Retsch Inc., Newtown, PA, USA) in 10-mL stainless steel jars with 10 mm stainless steel balls. Ground hair (50 mg) was added to 7-mL glass scintillation vials with 1.5 mL of methanol, before shaking at 100 rpm for 18 h at 30 °C on a shaker. After incubation, a commercial ELISA assay was used to determine cortisol concentration (Salimetrics LLC, State Collage, PA, USA). All intra- and inter-assay CV values were ≤ 10% and ≤ 15% respectively.

### Growth performance and carcass measurements

Steers (*n* = 40; 8 cannulated, 32 unaltered) were weighed every 28 d and on two consecutive days at the beginning and end of the experiment to estimate ADG. Body weights were reported as shrunk BW by multiplying BW by 0.96 to account for gut fill. Average daily gain was calculated by using the total weight gain from shrunk body weight and dividing it by the total number of days on feed. Feed efficiency (G:F) was calculated by dividing ADG by DMI.

Net energy for gain (NEg) was calculated from estimates of retained energy (EG, Mcal/d) based on the growth performance of yearling steers (NASEM, 2016): EG = 0.0493 × [MW × (478/650)]^0.75^ × ADG^1.097^, where the mean shrunk body weight (MW) = [(initial BW × 0.96 + final BW × 0.96)/2]. The net energy for maintenance (NEm) was determined as described by Zinn et al. (2002) and NEg was then estimated using the equation: NEg = 0.877 × NEm – 0.41.

At the end of the trial, intact steers (*n* = 32) were sent to Cargill Foods, High River, Alberta, Canada for processing. Cannulated steers (*n* = 8) were sent to Alberta Prairie Meats, Duchess, Alberta for processing. Hot carcass weight (HCW), dressing percentage, back fat thickness, rib eye area (REA), saleable meat yield, and quality grade were determined for non-cannulated steers (*n* = 32) by qualified graders. Saleable meat yield (%) was used to estimate lean meat yield using the length, width, and fat cover of the longissimus dorsi between the 12th and 13th rib:

Lean meat yield (LMY) = 57.96 – 0.027 × (carcass weight, kg) + 0.202 × (rib eye area, cm^2^) – 0.703 × (back fat thickness, mm).

Marbling quality scores were determined using five levels of intramuscular fat; B = devoid; A = trace; AA = slight; AAA = small to moderate; and prime = abundant or greater (CBGA 2009). Livers were collected from all steers and assessed for the incidence and severity of abscesses using the Elanco Liver Score System (Elanco Animal Health, Greenfield, IN, USA). Liver scores were assigned at four different rankings: 0 = no abscesses present; A- = one or two abscesses present, or abscess scars observed; A = two to four medium abscesses (less than 2.5 cm in diameter); and A +  = one or more large, active abscesses (greater than 2.5 cm).

### Statistical analysis

Data were analyzed using the MIXED procedure of SAS (SAS Inst. Inc., Cary, NC, USA). Diets were included as fixed effects and individual steer was considered a random effect and the experimental unit for all parameters. Protozoa, VFA, NH_3_-N, and blood parameters were analyzed using period as a repeated measure with day 0 included as a covariate for each variable. The minimum value of Akaike’s information criterion (AIC) was used to select among the covariance structures of unstructured, compound symmetry, heterogeneous compound symmetry, autoregressive, heterogeneous autoregressive, Toeplitz, heterogeneous banded Toeplitz, and ante-dependence structures for each parameter. Data from pH loggers, liver abscesses, and carcass quality traits were analyzed using the GLIMMIX procedure (SAS Inst. Inc., Cary, NC, USA). The sums of squares were further partitioned by orthogonal contrasts to analyze overall differences between CON and MYC treatments, as well as differences between CON-L and MYC-L, and MYC-L and MYC-H treatments. Means were declared significant at *P* ≤ 0.05, and tendencies discussed at 0.05 < *P* ≤ 0.10.

## Results

### Diet composition and mycotoxin contamination levels

Dietary mycotoxin concentrations were based on CFIA maximum allowable limits of 5 mg/kg for DON in cattle diets. The MYC-L diets were formulated to contain 5 mg/kg of DON, which is the upper limit according to CFIA guidelines. Previous research showed that dairy cattle were insensitive to 12 mg DON/kg DM (Charmley et al. 1993). As a result, the MYC-H diet was formulated to exceed CFIA regulations and contained 10 mg/kg DON to further assess the sensitivity of feedlot cattle to this mycotoxin. In addition to DON, wheat screenings also contained EAs, with EA inclusion rates for MYC-L and MYC-H diets being 2.1 mg/kg and 4.2 mg/kg, respectively. Compared to CFIA guidelines, the MYC-L group was within the allowable limit of 2 to 3 mg/kg, whereas recommended levels of EAs were exceeded in MYC-H.

### Intake, digestibility, and growth performance

The intake of DM, starch, CP, NDF, and ADF were lower (*P* < 0.001) in MYC steers compared to CON steers (Table 2). The MYC-L steers had lower (*P* < 0.001) DM, starch, and CP intake than CON-L steers, with a decrease of 1.47 kg/day, 0.77 kg/day, and 0.20 kg/day, respectively. There were no differences (*P* ≥ 0.12) between CON-L and MYC-L steers in NDF and ADF intake. Increasing dietary MYC content further decreased intake, as intake of MYC-H steers compared to MYC-L steers was lower (*P* < 0.001) by 2.09 kg/day, 1.04 kg/day, 0.39 kg/day, and 0.17 kg/day for DM, starch, CP, and NDF, respectively. Digestibility of DM, CP, and starch were greater (*P* ≤ 0.002) for MYC steers compared to CON steers, with this increased digestibility still apparent in MYC-L vs CON-L steers. No differences were noted in digestibility between MYC (L vs. H) treatments.

**Table 2:** Feed intake and apparent total tract nutrient digestibility for feedlot steers fed control diets or mycotoxin contaminated diets (*n* = 10 steers per treatment)

	CON		MYC			*P*-Value		
Item	Low	High	Low	High	SEM	CON vs MYC	CON-L vs MYC-L	MYC-L vs MYC-H
Intake, kg/day								
Dry matter	9.85	9.67	8.38	6.29	0.22	< 0.001	< 0.001	< 0.001
Starch	4.46	4.14	3.69	2.65	0.12	< 0.001	< 0.001	< 0.001
Crude protein	1.63	1.61	1.43	1.04	0.04	< 0.001	< 0.001	< 0.001
Neutral detergent fiber	1.10	1.28	1.05	0.88	0.03	< 0.001	0.40	< 0.001
Acid detergent fiber	0.48	0.61	0.44	0.42	0.04	< 0.001	0.12	0.40
Digestibility, %								
Dry matter	82.4	82.5	84.7	84.9	0.38	0.002	0.03	0.82
Crude protein	83.1	83.0	85.8	85.2	0.35	< 0.001	0.003	0.47
Starch	97.9	98.3	99.0	99.2	0.14	< 0.001	0.002	0.60

By design, initial BW did not differ among treatments at the start of the experiment (Table 3). Overall final shrunk BW (*P* < 0.001), ADG (*P* < 0.001), and G:F (*P* = 0.001) were lower for MYC steers compared to CON steers. The MYC-L steers were lighter (*P* < 0.001) and had a lower ADG (*P* < 0.001) than CON-L steers. Steers fed MYC-H were lighter (*P* = 0.005) and had lower ADG (*P* = 0.003) than steers fed MYC-L. Although G:F tended to be lower (*P* = 0.08) for MYC-L than CON-L steers, it did not differ (*P* = 0.10) between MYC-L and MYC-H steers. Overall net energy of gain (NEg) was lower (*P* < 0.001) for MYC steers compared to CON steers. Steers fed the MYC-L diet also had a lower NEg (*P* = 0.003) compared to CON-L steers. However, NEg did not differ between MYC-L and MYC-H steers (*P* = 0.13).

**Table 3:** Growth performance of feedlot steers fed a wheat-based finishing diet with and without mycotoxins (*n* = 10 steers, per treatment)

	CON		MYC			*P*-Value		
Item	Low	High	Low	High	SEM	CON vs MYC	CON-L vs MYC-L	MYC-L vs MYC-H
Shrunk initial BW^a^, kg	453	448	450	449	5.82	0.93	0.86	0.94
Shrunk final BW, kg	670	665	624	561^c^	9.95	< 0.001	0.001	0.005
ADG, kg/day	1.91	1.89	1.35	0.85	0.07	< 0.001	< 0.001	0.001
Gain:Feed	0.195	0.197	0.164	0.134	0.01	< 0.001	0.080	0.10
NEg^b^, Mcal/kg DM	1.91	1.93	1.56	1.39	0.03	< 0.001	0.003	0.13

^a^Body Weight

^b^Net energy for gain

### Ruminal ammonia, VFA, and protozoa

The MYC steers had lower total VFA concentrations (*P* = 0.03) compared to CON steers (Table 4). The MYC-L steers also tended to have lower total VFA concentrations (*P* = 0.06) than CON-L steers, with no difference between MYC-L and MYC-H steers (*P* = 0.26). Ammonia-N concentration, protozoa counts, and individual VFA proportions were not affected (*P* > 0.10) by inclusion of mycotoxins in the diet.

**Table 4:** Ruminal ammonia and VFA in feedlot steers fed a wheat-based finishing diet with and without two levels of mycotoxins (*n* = 2 steers per treatment)

	CON		MYC			*P*-Value		
Item	Low	High	Low	High	SEM	CON vs MYC	CON-L vs MYC-L	MYC-L vs MYC-H
Total VFA, m*M*	120	96.9	90.5	76.5	4.99	0.03	0.06	0.26
Ammonia-N, m*M*	16.0	13.0	13.9	11.6	0.64	0.24	0.32	0.26
Protozoa, *n* × 10^5^	5.60	5.64	5.62	5.64	0.01	0.57	0.56	0.30
VFA, mol/100 mol								
Acetate	49.9	50.5	56.4	41.1	0.90	0.73	0.31	0.22
Propionate	34.9	32.1	29.5	32.7	0.87	0.42	0.23	0.59
Butyrate	8.22	10.4	10.1	10.2	0.47	0.53	0.32	0.99
Isobutyrate	1.36	1.76	1.87	1.79	0.07	0.28	0.17	0.24
Valerate	3.16	4.16	2.60	3.22	0.27	0.65	0.70	0.62
Caproate	0.46	0.49	0.23	0.70	0.06	0.32	0.15	0.19
Acetate:Propionate	1.28	1.40	1.44	1.43	0.04	0.46	0.39	0.95

### Ruminal pH

Mean, min, max, and standard deviation (SD) of the mean were unaffected (*P* ≥ 0.25) by MYC (Table 5). Durations of pH < 6.0, 5.6, and 5.2 were shorter (*P* < 0.001) for MYC than CON steers, with shorter durations (*P* ≤ 0.004) also reported for MYC-L compared to CON-L groups. As the magnitude of contamination increased (MYC-L steers vs. MYC-H steer), durations below each pH threshold were decreased (*P* < 0.001). The AUC for MYC steers was smaller (*P* < 0.001) for both pH thresholds of 6.0 and 5.6 compared to CON steers. The MYC-L steers also had a smaller (*P* < 0.001) AUC compared to CON-L steers for both thresholds. Feeding higher concentrations of mycotoxins (MYC-H vs MYC-L) led to a decrease (*P* ≤ 0.03) in AUC for both pH thresholds (< 6.0, < 5.6). The AUC/kg of DM for pH < 6.0 and 5.6 was smaller (*P* < 0.001) for MYC steers compared to CON steers. Increased concentration of MYC in the diet also led to a decrease (*P* ≤ 0.01) in AUC/kg of DMI.

**Table 5:** Ruminal pH of feedlot steers fed a wheat-based finishing diet with and without two levels of mycotoxins (*n* = 2 steers per treatment)

	CON		MYC			*P*-Value		
Item	Low	High	Low	High	SEM	CON vs MYC	CON-L vs MYC-L	MYC-L vs MYC-H
Ruminal pH								
Mean	5.83	5.86	6.15	6.39	-	0.25	0.54	0.66
Minimum	5.13	5.13	5.31	5.49	-	0.43	0.70	0.72
Maximum	6.72	6.86	7.00	7.05	-	0.54	0.61	0.94
SD of mean	0.43	0.52	0.49	0.44	-	0.95	0.63	0.69
Duration of pH, h/d								
< 6.0	15.7	14.8	10.6	5.21	-	< 0.001	< 0.001	< 0.001
< 5.6	8.95	9.44	4.36	2.01	-	< 0.001	< 0.001	< 0.001
< 5.2	1.49	2.69	0.81^c^	0.09	-	< 0.001	0.004	< 0.001
AUC^1^, pH × h/d								
< 6.0	7.19	7.74	3.80	1.75	-	< 0.001	< 0.001	< 0.001
< 5.6	2.18	2.76	0.73	0.37	-	< 0.001	< 0.001	0.03
AUC/kg DMI, pH × min								
< 6.0	39.0	43.9	24.8	17.0	-	< 0.001	< 0.001	< 0.001
< 5.6	11.6	15.6	4.78	3.64	-	< 0.001	< 0.001	0.01

### Hair cortisol and blood parameters

There were no differences in hair cortisol concentrations (*P* = 0.72) between MYC and CON steers. However, MYC-L steers tended to have a lower (*P* = 0.07) hair cortisol concentration than CON-L steers (Table 6). A greater dose of MYC did not affect (*P* = 0.39) hair cortisol concentration (MYC-L vs. MYC-H steers).

**Table 6:** Hair cortisol, liver parameters, blood cell counts, reactive oxygen species (ROS), and cytokines of steers fed wheat-based feedlot diets with and without mycotoxins (*n* = 10 steers per treatment)

	CON		MYC			*P*-Value		
Item	Low	High	Low	High	SEM	CON vs MYC	CON-L vs MYC-L	MYC-L vs MYC-H
Hair cortisol, pg/mg	3.88	3.18	3.35	3.56	0.06	0.72	0.07	0.46
Liver parameters								
AST, U/L	104	120	88.9	110	3.00	0.06	0.08	0.04
ALT, U/L	66.2	63.8	62.9	54.5	1.00	0.008	0.27	0.02
GGT, U/L	37.9	46.7	35.5	37.3	1.17	0.13	0.63	0.91
Albumin, g/L	31.3	31.6	30.6	29.5	0.34	0.08	0.50	0.11
Globulins, g/L	45.0	48.5	41.1	41.1	0.66	0.001	0.06	1.00
Albumin:Globulin	0.69	0.66	0.73	0.73	0.01	0.003	0.10	0.14
BUN, mmol/L	7.25	7.09	6.45	5.65	0.10	< 0.001	0.02	0.04
Blood cell count								
RBC, 10^6^/µL	9.06	9.22	9.32	9.37	0.06	0.09	0.13	0.07
WBC, 10^3^/µL	8.46	8.78	9.15	9.15	0.12	0.16	0.19	0.19
PLT, 10^3^/µL	286	284	249	256	3.51	< 0.001	0.003	0.53
LYM, 10^3^/µL	4.83	5.17	5.63	5.54	0.09	0.002	0.003	0.74
Reactive oxygen species								
Catalase, (nmol/min/mL)	0.119	0.106	0.106	0.104	0.003	0.57	0.33	0.95
Glutathione peroxidase, (nmol/min/mL)	78.6	72.6	97.6	91.6	1.70	< 0.001	< 0.001	0.24
Cytokines								
IL-6, (ng/mL)	4.77	4.62	4.58	4.72	0.08	0.78	0.35	0.49
IL-10, (ng/mL)	56.8	56.2	76.8	60.2	2.17	0.001	< 0.001	0.001
TNF-α, (ng/mL)	2.02	2.41	2.26	2.14	0.26	0.62	0.74	0.70

Of the liver parameters analyzed, MYC steers had lower (*P* < 0.008) blood ALT, globulin, and urea-N concentrations and higher A:G ratios (*P* = 0.003) compared to CON steers. Albumin and AST concentration tended to be lower (*P* ≥ 0.06) in MYC steers than CON steers. Furthermore, MYC-L steers had lower (*P* = 0.02) BUN concentrations than CON-L steers. The MYC-L steers also tended to have lower blood concentrations of AST (*P* = 0.08) and globulin (*P* = 0.06), and higher blood A:G ratios (*P* = 0.10) than CON-L steers. However, AST concentrations were higher (*P* = 0.04), whereas ALT and BUN concentrations were lower (*P* ≤ 0.04) for MYC-H compared to MYC-L steers.

Overall platelet count (PLT) was lower (*P* < 0.001) for MYC than CON steers. MYC-L steers also had a lower (*P* = 0.003) PLT count than CON-L steers. However, there were no differences (*P* = 0.53) in PLT between MYC-L and MYC-H steers. The MYC steers tended to have greater (*P* = 0.09) RBC counts than CON steers, with no differences (*P* = 0.13) noted between CON-L and MYC-L steers. However, MYC-L steers tended (*P* = 0.07) to have lower RBC counts compared to MYC-H steers. Overall, lymphocyte counts (LYM) were greater (*P* = 0.002) in MYC than CON steers, with MYC-L steers also having greater (*P* = 0.003) LYM counts compared to CON-L steers.

Feeding MYC infected wheat screenings led to an increase (*P* ≤ 0.001) in glutathione peroxidase activity (GPx) and interleukin-10 (IL-10) in steers. No differences (*P* = 0.24) were noted in GPx, between MYC-L and MYC-H steers. However, MYC-L steers had higher IL-10 than their MYC-H counterparts. The presence of MYC did not affect (*P* ≥ 0.57) catalase activity, IL-6, or TNF-α.

### Carcass characteristics and liver analysis

Addition of MYC to wheat-based feedlot diets led to a decrease (*P* < 0.001) in hot carcass weight and back fat thickness (Table 7). Steers fed the MYC-L diet had lower hot carcass weight (*P* = 0.003) and less back fat (*P* = 0.04) than steers fed the CON-L diet. The MYC-H steers also had lower (*P* = 0.02) carcass weight than MYC-L steers. However, there was no difference in back fat thickness (*P* = 0.32) between MYC-L and MYC-H steers. There was also a tendency (*P* = 0.06) for MYC steers to have a smaller rib eye area (REA) compared to CON steers, with no differences (*P* ≥ 0.18) between CON-L and MYC-L steers or between MYC-L and MYC-H steers. However, steers fed mycotoxin-containing diets had more saleable meat and leaner carcasses (*P* < 0.01) than steers fed the CON diet. Lean meat yield in the MYC-L steers was also greater (*P* = 0.01) when compared to the CON-L steers, and there was a tendency (*P* = 0.09) for MYC-L steers to have more saleable meat than CON-L steers. There were no differences (*P* ≥ 0.37) in saleable and lean meat yield between MYC-L and MYC-H steers. Feeding of mycotoxin contaminated wheat screenings did not affect (*P* ≥ 0.13) overall dressing percentage, marbling, or incidence and severity of liver abscesses. However, MYC-L steers had a greater (*P* < 0.001) proportion of carcasses classified as AAA quality compared to CON-L steers.

**Table 7:** Carcass characteristics and incidence of liver abscesses in feedlot steers fed a wheat-based finishing diet with and without mycotoxins (*n* = 8 steers per treatment)

	CON		MYC			*P*-Value		
Item	Low	High	Low	High	SEM	CON vs MYC	CON-L vs MYC-L	MYC-L vs MYC-H
Hot carcass weight, kg	429	430	383	347	7.83	< 0.001	0.003	0.02
Dressing percentage	62.2	62.8	62.5	62.6	0.28	0.96	0.76	0.69
Back fat, mm	23.2	22.7	18.0	15.5	1.00	< 0.001	0.04	0.32
Rib eye area, cm^2^	101	97.6	94.9	91.0	1.67	0.06	0.18	0.40
Saleable meat yield, %	50.4	51.1	54.1	56.0	0.82	0.007	0.09	0.37
Lean meat yield^a^, %	46.7	47.4	52.8	53.4	0.96	< 0.001	0.01	0.79
Quality grade^b^, AAA%	100.0	75.0	87.5	75.0	-	0.53	< 0.001	0.30
Abscessed livers^c^, %	60.0	50.0	40.0	20.0	-	0.13	0.39	0.36
Severely abscessed livers, %	30.0	30.0	10.0	0.00	-	0.97	0.18	0.98

^a^Lean yield = 57.34 + (0.212 × REA [cm2]) – (0.81 × grade fat [mm]) – (0.032 × HCW [kg])

^b^Canada grade AAA (approximately equivalent to United States Department of Agriculture choice); marbling score 500

^c^Severely abscessed livers = livers classified as A+ (one or more large active abscesses greater than 2.5 cm diameter with inflammation of surrounding tissue) were considered severely abscessed

## Discussion

Mycotoxins are secondary metabolites of low molecular weight that are toxic to a variety of livestock species. Because they are not proteins like most bacterial toxins, mycotoxins are not recognized as antigens by the immune system and therefore do not trigger an antibody mediated reaction to combat their presence (Rodrigues 2014). Ruminants are generally considered to be more resistant to mycotoxins than non-ruminants, like poultry and swine (Jouany et al. 2009). This reflects the ability of the rumen microbiota to bio-convert most mycotoxins to less toxic intermediates. However, most of the studies investigating the sensitivity of ruminants to multiple mycotoxins have not investigated ergot alkaloids. Studies that have been conducted suggest that ruminants may be more sensitive than non-ruminants to ergot alkaloids (Coufal-Majewski et al. 2017; Stanford et al. 2022; Sarich et al. 2023). This could potentially be due to the absorption potential of the ruminant forestomach (Hill et al. 2001) along with a limited ability of rumen microbiota to detoxify EAs (Klotz 2015).

While DON has a reputation of being well tolerated by ruminants compared to EAs, studies investigating fusarium mycotoxin mixtures with DON indicate that it may elicit negative immune-modulatory effects in cattle when present with other contaminants (Gallo et al. 2022). Deoxynivalenol can interact with the 60S ribosomal subunit, disrupting protein synthesis, thereby restricting cell proliferation and potentially compromising immunity (Novak et al. 2018). Roberts et al. (2021) showed that beef cattle fed a combination of DON and B class FUM (B1, B2, and B3) for 35 days at 1.7 mg/kg and 3.5 mg/kg of diet DM respectively, experienced reduced protein digestibility, and immune function. To date we are unaware of any additional studies that have assessed the impacts of feeding diets containing mixtures of DON and EAs to ruminants.

### Intake, digestibility, and growth performance

A mixture of DON and EAs reduced the DMI of feedlot steers when included at the limits set by CFIA (CFIA 2017a). Intake of DM further decreased as the dietary mycotoxin content was increased to double the maximum allowable limits, with MYC-L and MYC-H steers consuming 14.9% and 35.0% less than CON steers. This reduction could be explained by the interaction of EAs with serotonin receptors, specifically 5-HT (5-hydroxytryptamine), which can supress the appetite of cattle (Valente et al. 2021). The ergoline ring has a structure similar to serotonin, dopamine, and norepinephrine (Strickland et al. 2011), enabling it to bind to the same biogenic receptors. Valente et al. (2020) fed Holstein steers either 0 (control), 0.86, or 1.28 mg of EAs/kg of diet DM derived from endophyte infected tall fescue and reported a 15.1% and 27.1% decrease in DMI at inclusion levels 0.86 mg/kg and 1.28 mg/kg, respectively. Similarly, Riccioni (2017), administered EAs (20 µg ergovaline + ergovalinine/kg BW) to steers and observed a decline in DMI compared to steers that did not receive alkaloids. Compared to previous research conducted with EAs in ruminants, the majority of studies report that DON has little to no impact on DMI (Seeling and Dänicke 2005). However, a study conducted by Winkler et al. (2016) reported that feeding varying concentrations of DON (0.36, 3.01, 5.66, and 8.31 mg/kg of DM) to growing Holstein bulls increased DMI as the level of contamination increased. Winkler et al. (2016) concluded that due to the interference of DON with immunological processes, the bulls fed higher DON concentrations had a greater requirement for metabolizable energy and protein. Based on results from previous studies where DON and EAs were fed individually, it is likely that EA inclusion was the primary driving force behind the decrease in DMI in the current study, however more research is needed to discern for certain the mycotoxin responsible. EAs also seemed to alleviate any DON mediated increases in intake as observed in previous studies where only DON was administered to cattle. After feeding only EAs at a concentration of 2.0 mg/kg to steers, Reynolds et al. (2024) reported a 7.5% decrease in DMI. Similarly, Sarich et al. (2023) reported DMI reduction up to 11.5% when steers where fed a finishing diet with 3.0 mg EAs/kg of diet DM. These were lower than the 13.2% decrease in DMI in our study with the MYC-L diet (2.1 mg/kg EAs; 5.0 mg/kg DON). These comparisons also suggest that there may be interactions between the two mycotoxins that further harm growth performance.

The increase in apparent total tract digestibility observed with mycotoxins is likely a reflection of decreased intake. As intake decreases, diet digestibility generally increases due to increased ruminal retention time (Ferrell 1993). However, Klotz (2015) reported that EAs have variable impacts on digestibility in cattle. Some studies reported increased diet digestibility in Holstein cows and steers (Goetsch et al. 1987; Schumann et al. 2008; Koontz et al. 2014), while others reported decreased digestibility in beef steers, heifers, and sheep (Aldrich et al. 1993; Westendorf et al. 1993; Matthews et al. 2005). Some in vitro studies have suggested that EAs may have negative effects on ruminal starch degrading *Ruminobacter* species (Klieve et al. 2012; Hua et al. 2021; Ma et al. 2021), which could contribute to a decrease in the digestibility of high grain diets. However, Sarich et al. (2022) showed that a high level of EAs (20 mg/kg of diet DM) had very limited to no impact on digestion or bacterial communities in an artificial rumen (RUSITEC). This finding further supports the idea that reduced DMI in MYC fed steers ultimately resulted in increased diet digestibility compared to CON steers rather than alterations to the rumen microbial communities (Sarich et al. 2022). The variation in DM intake responses observed among studies when EAs were fed at similar dietary concentrations also reinforces the difficulty in predicting detrimental levels of EAs. This variation may reflect the vast array of alkaloid profiles that can occur within sclerotia including epimers that differ in toxicity (Orlando et al. 2017). Furthermore, the sensitivity of individuals to EAs may also differ due to differences in liver enzyme activities and rumen microbial profiles (Coufal-Majewski et al. 2016). It is also important to note that *Fusarium* infections produce and release extracellular plant-cell-wall-degrading enzymes and proteases when colonizing a host plant (Alconada et al. 2019), which could potentially cause “pre-digestion” increasing digestibility of the screenings (Goyarts et al. 2005). However, research investigating the effect of *Fusarium* infections on nutrient digestion have been inconsistent (Dänicke et al. 2004) and levels of screenings in the diet in the current study may have been too low to detect any differences in digestibility of DON contaminated wheat screenings vs those that were not.

The drastic reduction in DMI in MYC steers was also reflected in their growth, as they were unable to fully meet their energy and protein needs. Therefore, ADG was reduced by 29% and 55%, and G:F by 16% and 32% for MYC-L and MYC-H steers respectively, compared to CON steers. Furthermore, shrunk final body weight and NEg were also lower in MYC steers due to compromised DMI and ADG. Stanford et al. (2022) fed backgrounding beef steers diets containing 0, or 1.6 mg/kg EAs and reported no impact on animal performance. Reynolds et al. (2024) reported a 25% decrease in ADG and an 8.4% decrease in G:F for steers fed diets containing 2 mg EA/kg diet DM. This was smaller than the decrease in ADG and G:F we observed for MYC-L steers. Sarich et al. (2023) observed decreased growth performance in steers fed a TMR containing 3.97 mg of EAs/kg of DM, a concentration of EA similar to MYC-H (4.2 mg EAs/kg of DM; 10 mg DON/kg of DM). However, performance losses reported by Sarich et al. (2023) were not as severe as those observed in our study. These results suggest that there could be additive effects between DON and EAs, leading to a more severe impact on cattle performance, even when both mycotoxins are within CFIA allowable limits.

### Rumen function

Inclusion of DON and EAs contaminated wheat screenings in the diet decreased total VFA concentrations within the rumen compared to CON diets. The decrease in DMI for steers fed mycotoxin diets reduced the amount of fermentable carbohydrates available to rumen microbes to produce VFA (Schären et al. 2016). This is further supported by the lack of changes in VFA proportions, suggesting that EAs had little effect on rumen microbial populations, results which agree with Sarich et al. (2022). Acetate, propionate, and butyrate are produced as result of the fermentation of carbohydrates by the rumen microbial community and are the main source of energy for the ruminant host (Zhang et al. 2022). This reduction in energy absorption due to lower VFA concentration and production could further explain why MYC steers exhibited such a drastic decrease in growth performance. An in vitro study investigated the effects of increasing DON from 5 mg/kg to 20 mg/kg and found that fermentation and total VFA production declined (Zhang et al. 2022). This suggests that rumen microbial activity may be influenced by high concentrations of DON. Ma et al. (2021) used 0%, 25%, 50%, 75%, or 100% endophyte infected horse grass (*Achnatherum inebrians)* to assess the impact of EAs on in vitro fermentation. They also found that total VFA concentrations decreased with increasing levels of endophyte infected grass. When we compare these results to our current study, it is not possible to specifically determine which mycotoxin was having a negative effect on VFA production. To help reduce the effects of mycotoxins in livestock, binders can be used to limit their bioavailability, thereby reducing their negative impacts (Coufal-Majewski et al. 2016). Sarich et al. (2022) conducted an in vitro study using a control (0.05 mg/kg EAs), 20.95 mg/kg EAs, and EAs at 25 mg/kg with a mycotoxin binder. In that study, EAs had a detrimental impact on ruminal microbial diversity, decreasing the Verrucomicrobiota and cellulose degradation (Gharechahi et al. 2020), responses that were negated by the addition of a mycotoxin binder.

Steers fed MYC diets experienced a shorter duration below all ruminal pH thresholds (< 6.0, 5.6, 5.2) compared to CON diets, a reflection of the lower DMI of steers consuming mycotoxins. These results suggest that MYC steers experienced a less acidotic ruminal environment compared to CON steers. Although the decrease in ruminal pH was less severe for MYC steers than CON steers, any benefits as result of improved rumen function did not offset the reduction in growth performance due to lower DM intake.

### Physiological impacts and incidence of liver abscesses

Mycotoxins occurring in feed have been well documented to have negative physiological effects on livestock (Santos Pereira et al. 2019). With an increasing incidence of subclinical mycotoxicosis in cattle, hematological analyses are becoming increasingly important as a diagnostic technique (Abramowicz et al. 2019). While no differences were noted in RBC and WBC counts, platelet counts were lower in steers exposed to MYC. Reduced platelet counts have been reported in bovine, ovine, and caprine species fed feeds contaminated with trichothecene mycotoxins (Jones and Allison 2007; Kocatürk et al. 2010; Bell 2011; Roland et al. 2014). Deoxynivalenol is a mycotoxin with immunomodulatory properties that has been linked to thrombocytopenia (Nugent et al. 2009), as opposed to EAs which are more often linked to thrombosis (Coufal-Majewski 2017). However, most studies conducted with DON have reported thrombocytopenia in humans, domesticated pets, and piglets (Parent-Massin 2004; Pierron et al. 2018) with little to no data available for ruminants. The lack of research on thrombocytopenia/reduced platelet count reported in ruminants is likely due to their ability to detoxify DON (Seeling et al. 2006). However, in the present study, feeding diets containing DON and EAs led to a decrease in platelet count. Therefore, it is possible that the increased physiological strain due to the presence EAs in conjunction with DON may cause cattle to be more susceptible to the disruptive effects of DON on protein synthesis.

We also observed an increase in lymphocyte counts in cattle consuming DON and EAs, supporting their role in altering immune function in cattle. Dänicke et al. (2018) attempted to assess the impact of DON and zearalenone (ZEN) on immunity by measuring antibody responses in Holstein bulls either vaccinated, or unvaccinated against bovine viral diarrhea virus (BVDV). Bulls consuming 5.66 mg DON/kg DM and 0.48 mg ZEN/kg DM had the highest immune response, while bulls fed 8.31 mg/kg DM DON and 0.69 mg/kg ZEN had the lowest immune response based on CD4 and CD8 lymphocyte counts. Because DON interrupts protein synthesis by interfering with the 60S ribosomal subunit, decreases in quick proliferating immune cells would be expected. However, as we observed the opposite response with a mixture of DON and EAs, EAs maybe the main factor influencing lymphocyte counts in the mixture as opposed to DON. Stanford et al. (2022) observed that ergot alkaloids averaging 1.6 mg/kg of DM in the diets of backgrounding beef steers did not alter lymphocyte counts but did increase them as a proportion of total white blood cells. Rahimabadi et al. (2022) reported that lymphocyte count was increased in feedlot cattle suffering from ergot toxicosis, although the EAs were not directly measured. However, given the observed clinical symptoms (i.e., tail loss, ear loss, recumbency, and death), the levels of ergot alkaloids must have been extremely high. When comparing results for MYC-L steers (5.0 mg DON and 2.2 mg EAs per kg/DM) to the 1.6 mg/kg EAs administered to beef steers by Stanford et al. (2022), it appears that when mixed with DON, EAs were more detrimental to the immune response than when consumed alone.

Liver function markers ALT and globulins were lower, and AST tended to be lower in MYC steers than CON steers. This may be a reflection of the greater acidity in the rumen of CON compared to MYC steers. Previous research with dairy cows and goats has reported an increase in ALT, AST, and globulins in the serum of ruminants experiencing both subclinical and clinical acidosis (Nasr et al. 2017; Saravanan et al. 2021; Morar et al. 2022).

While in the present study there were no differences in liver abscesses between treatments, incidence of total and severe liver abscesses were 30% and 5% for MYC steers and 55% and 30% for CON steers, respectively. Acidosis has been generally accepted as a predisposing factor to liver abscesses, with lesions in the rumen allowing bacteria to be absorbed from the rumen into the portal vein and infect the liver (Nagaraja and Chengappa 1998). Lysed liver cells release AST and ALT into the blood stream, causing elevated levels in serum (Macdonald et al. 2017; Ahmed 2020). This idea is further supported by the higher DMI, increased VFA production, and lower ruminal pH of CON steers, compared to the MYC steers. The decrease in globulins in MYC steers would also agree with the lower levels of ALT as a result of fewer abscesses than CON steers. Because immunoglobulins are proteins produced by the immune system as a result of an infection, the occurrence of liver abscesses would be expected to increase levels of serum globulins (Herrick et al. 2020).

While it is likely that the mixture of DON and EAs in the current experiment did not have an effect on liver function, previous studies in both pigs and rats have demonstrated that DON can impair liver function and cause apoptosis (Bai et al. 2021; Li et al. 2022). Few studies have investigated the effect of EAs on liver function. Dänicke and Diers (2013) investigated the possible effects of EAs on the liver in piglets and reported no hepatocellular damage. However, research conducted in rats concluded that EAs did increase glycogen storage and lipogenesis in the liver, possibly due to the dopaminergic properties of ergocryptine (Janssen et al. 2000a,b). Blood urea nitrogen (BUN) was also elevated in CON steers, likely reflecting their greater CP intake (Gleghorn et al. 2004).

Many studies have highlighted the ability of mycotoxins to induce oxidative stress (Doi and Uetsuka 2011; Omar 2013; Da Silva et al. 2018; Liang and Wang 2023); but few have investigated the effects of mixtures (Sharma and Patial 2021). Glutathione peroxidase activity (GPx) was chosen to measure oxidative stress as it is located in the cytosol and in the mitochondrial matrix of nearly all mammalian tissues. In our study, GPx was increased in steers exposed to a mixture of DON and EA. Currently, there is little data available on GPx concentrations in the serum or plasma of ruminants exposed to DON. However, Wang et al. (2019) observed that DON caused an increase in reactive oxygen species in bovine mammary epithelial cells, and Hou et al. (2013) reported elevated GPx in the serum of mice fed DON contaminated diets. While there have been studies conducted investigating the effect of high DON levels in dairy cattle (Côté et al. 1986; Charmley et al. 1993; Ingalls 1996), they did not assess oxidative stress. In the case of EAs, decreases in GPx activity in the liver have been reported by Settivari et al. (2008) in which rats were fed endophyte infected tall fescue.

Interleukin-10 is an important cytokine with anti-inflammatory properties (Sabat et al. 2010), but the effect of mycotoxins on its production in cattle has not been studied. Döll et al. (2009) reported no effect of DON on IL-10 production in porcine hepatocytes but did observe elevated levels of IL-10 upon exposure to lipopolysaccharides. Similarly, Bracarense et al. (2012) reported no differences in IL-10 in piglets consuming diets containing DON. However, when DON was present with FUM (FB), there was an increase in IL-10 in the jejunum of piglets. Data on the effects of EAs on cytokines in cattle are also scarce. Poole (2019) fed endophyte infected tall fescue (185 µg ergovaline/kg of BW) to steers and observed increases in most anti-inflammatory cytokines (IL-2, IL-15, IL-21, and IL-1-F5), except for IL-4. While their study did not measure IL-10, our study suggests that EAs may also elevate the levels of this cytokine. In another study, Hanneman (2018) fed seeds of endophyte infected tall fescue to horses (3.7 µg EA/kg) and observed no changes in IL-10. When combining the results from both DON and EAs studies, EAs may be the main mycotoxin responsible for elevated IL-10. However, more research is needed to discern which mycotoxin is the root cause of the responses recorded.

### Carcass traits

The reduction in DMI with inclusion of DON and EAs in the diet of steers resulted in reduced shrunk final BW and hot carcass weights (HCW). The results of the current study are in agreement with Realini et al. (2005), who showed that cattle grazing endophyte infected tall fescue with high EAs had decreased ADG, and lighter carcasses compared to control cattle. Reynolds et al. (2024) and Sarich et al. (2023) both fed EA-contaminated grain to feedlot cattle and made similar observations. Furthermore, because steers were not meeting their energy requirements for growth and fat deposition, MYC steers had decreased backfat thickness compared to CON steers. Consequently, carcasses from MYC steers were leaner than from CON steers, resulting in a higher proportion of saleable meat.

In conclusion, results from this study demonstrated that wheat grain contaminated with both DON and EAs negatively impacted growth performance and health status of feedlot steers. However, further studies would be needed to determine the degree that EAs alone, or a mixture of DON and EAs were responsible for the current results. This study highlights the importance of feed testing ingredients for mycotoxins prior to their incorporation into a TMR, and that CFIA limits for DON and EAs may be too high when the mycotoxins are co-occurring in feed as the MYC-L diet was formulated to be under those limits, but still resulted in a substantial reduction in growth performance. The results also raise the question of whether or not mycotoxins can interact with each another, and if so, what is the nature of these interactions. Future studies would benefit from having similar feed sources contaminated with only DON or EAs to help distinguish which mycotoxin is responsible for each response. However, due to the nature of mycotoxins often occurring together, this could make procuring of samples for experimentation could be difficult. Information from this study can be used to aid in the evaluation of CFIA maximum allowable guidelines for mycotoxins, while also helping to ensure the welfare of cattle for stock producers.

## Data Availability

All data supporting the findings of this study are available within the paper, raw data prior to statistical analysis are available from the corresponding author upon reasonable request.

## Abbreviations

ADF: Acid detergent fibre
ADG: Average daily gains
AG: Albumin to globulin ratio
AIC: Akaike’s information criterion
ALT: Alanine aminotransferase
AP: Acetate to propionate ratio
AST: Aspartate aminotransferase
AUC: Area under the curve
BUN: Blood urea nitrogen
BW: Body weight
CBC: Complete blood cell count
CCAC: Canadian Council on Animal Care
CFIA: Canadian Food Inspection Agency
CON: Control
CP: Crude protein
DM: Dry matter
DMI: Dry matter intake
DOM-1: De-epoxy DON
DON: Deoxynivalenol
EAs: Ergot alkaloids
FB_1_: Fumonisins B_1_
Fg: *Fusarium graminearum*
FHB: Fusarium head blight
GF: Gain to feed
GGT: Gamma-glutamyl transferase
GPx: Glutathione peroxidase activity
HCW: Hot carcass weight
LMY: Lean meat yield
LPS: Lipopolysaccharides
LYM: Lymphocyte counts
NDF: Neutral detergent fibre
NEg: Net energy of gain
NH3-N: Ammonia
PLT: Platelet counts
RBC: Red blood cell count
REA: Rib eye area
SARA: Subacute ruminal acidosis
TMR: Total mixed ration
VFA: Volatile fatty acids
WBC: White blood cell count
ZEN: Zearalenone
